# Maternal Protein Restriction Differentially Alters the Expression of AQP1, AQP9 and VEGFr-2 in the Epididymis of Rat Offspring

**DOI:** 10.3390/ijms20030469

**Published:** 2019-01-22

**Authors:** Marilia Martins Cavariani, Talita de Mello Santos, Dhrielly Natalia Pereira, Luiz Gustavo de Almeida Chuffa, Patricia Fernanda Felipe Pinheiro, Wellerson Rodrigo Scarano, Raquel Fantin Domeniconi

**Affiliations:** 1 Department of Anatomy, Institute of Biosciences, São Paulo State University-UNESP, 18618-970 Botucatu-SP, Brazil; talita_mellosantos@yahoo.com.br (T.d.M.S.); dhrielly.p@gmail.com (D.N.P.); luiz-gustavo.chuffa@unesp.br (L.G.d.A.C.); patricia.ff.pinheiro@unesp.br (P.F.F.P.); raquel.domeniconi@unesp.br (R.F.D.); 2 Department of Morphology, Institute of Biosciences, São Paulo State University-UNESP, 18618-970 Botucatu-SP, Brazil; scarano@ibb.unesp.br

**Keywords:** epididymis, protein restriction, AQP1, AQP9, VEGFr-2, testosterone, aldosterone

## Abstract

Background: Maternal protein restriction causes sperm alterations in the offspring, most of which are associated with epididymal functions. Because fluid reabsorption/secretion dynamics in the epididymal environment play important roles in the process of sperm maturation and concentration, we investigated the effects of maternal protein restriction on the expression of aquaporins (AQP1 and AQP9), vascular endothelial growth factor (VEGFa), and its receptor VEGFr-2 in different stages of postnatal epididymal development. Methods: Pregnant rats were divided into groups that received normoprotein (17% protein) and low-protein diets (6% protein) during gestation and lactation. After weaning, male rats only received the standard diet and were euthanized at the predetermined ages of 21, 44 and 120 days. Results: Maternal protein restriction decreased AQP1 and AQP9 expression in the initial segment and caput epididymis compared to the increased expression of these proteins observed in the corpus and cauda at all ages. Although protein restriction reduced the microvasculature density (MVD) on postnatal day (PND) 21 and 44, the MVD was unaltered on PND 120. Conclusions: Maternal protein restriction changed the structure or function of the offspring’s epididymis, specifically by affecting fluid dynamics and vasculogenesis in important stages of epididymis development.

## 1. Introduction

The developing fetus depends on the mother and the maternal environment to supply their nutritional needs. The components and quality of the maternal diet during critical developmental periods have been reported to influence the offspring genome in the uterus [1,2]. The epigenetic changes occurring in early life can permanently alter the phenotype of the adult organism, making it susceptible to a range of diseases that characterize metabolic syndromes, such as type 2 diabetes, hypertension, and coronary heart disease [3,4,5,6,7,8].

During pregnancy, adequate protein intake is recommended to ensure that the additional nitrogen demands of both mother and fetus are met since an increase in protein turnover to meet the requirements for rapid embryo growth occurs at this stage [9]. Based on these findings, the protein restriction model is one of the most well-characterized models of early growth restriction [6,10,11].

Relatively few epidemiological and experimental studies have examined the effects of a maternal low-protein diet on the male reproductive function compared to studies of the metabolic disturbances. However, this experimental model causes sperm alterations in male offspring, most of which are associated with epididymal functions, such as sperm motility, viability, and concentration [12,13,14,15].

The epididymis is a convoluted duct whose primary function is to transport the spermatozoa from the testis to the vas deferens. The main function of this organ occurs during sperm transit through the highly specialized lumen of epididymis; it is responsible for the concentration, maturation, storage, and protection of spermatozoa [16,17,18,19]. Important changes in the epididymal luminal fluid are induced by water reabsorption, which increases sperm and protein concentrations in the intraluminal environment and is possibly associated with the first modifications of the spermatozoa [20].

Aquaporins (AQP) are transmembrane proteins involved in the fluid reabsorption/secretion dynamics in the epididymal intraluminal environment, playing a pivotal role in the process of sperm maturation and concentration [21]. Six members of this family of proteins have been identified in the epididymis, including AQP9 and AQP1 [22,23]. AQP9 is continuously expressed in the apical region of the principal cells of the initial segment, caput, corpus, and cauda epididymis, while AQP1 is absent in epithelial cells of the epididymis and is expressed at high levels in the adjacent smooth muscle and endothelial cells of the organ’s vascular channels [23,24,25,26].

In addition to AQPs, aldosterone also influences the absorption of liquids from the epididymal lumen by promoting Na^+^ uptake against the electrochemical gradient throughout the epididymis [27,28,29]. The mechanisms regulating metabolite, hormone, and nutrient transport, as well as the secretion and reabsorption of luminal fluid in the epididymal duct, are crucial to maintaining the specificity of the epididymal microenvironment and require a highly branched vasculature to provide adequate blood supply [30,31]. Vascular endothelial growth factor (VEGF) and its receptor isoform (VEGFr-2) affect the microenvironment of the epididymis and sperm maturation [32]. In addition, treatment of epididymal tissue with VEGF causes morphological changes associated with increased capillary permeability, suggesting that this factor is an important regulator of vascular permeability in the epididymis [33]. The mechanisms by which protein restriction at an early stage of development affects the epididymal function remain to be identified. Therefore, this study investigated the effects of maternal protein restriction on serum testosterone and aldosterone levels in rat offspring, as well as the expression of AQP1, AQP9, vascular endothelial growth factor (VEGF)a, and VEGFr-2 at different stages of postnatal epididymal development. 

## 2. Results

### 2.1. A Maternal Low-Protein Diet Promotes Changes in Testosterone and Aldosterone Levels in an Age-Dependent-Manner 

**Figure 1:**
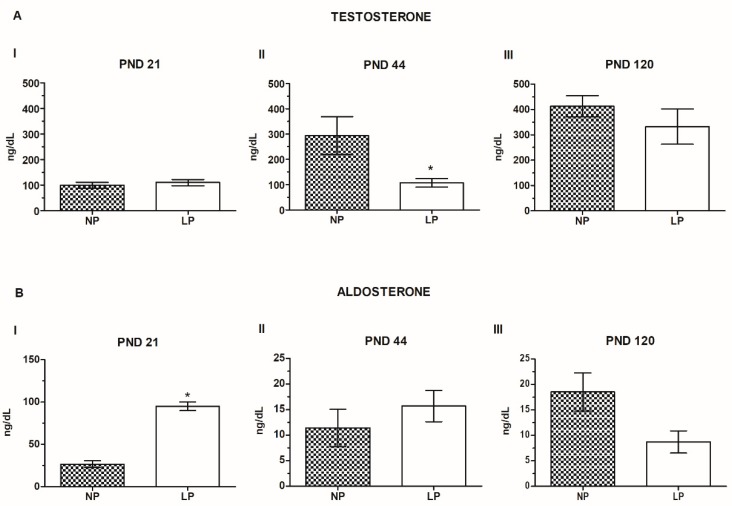
Serum hormones levels. (**A**) Plasma testosterone levels (ng/dL) at postnatal day (PND) 21 (**I**), PND 44 (**II**) and PND 120 (**III**), * *p* < 0.05 compared with the normoprotein (NP) group. (**B**) Plasma aldosterone levels (ng/dL) at PND 21 (**I**), PND 44 (**II**) and PND 120 (**III**), * *p* < 0.05 compared with the NP group. T-tests and the Mann-Whitney tests were used to assess the significance of the differences in parametric and nonparametric data, respectively.

Maternal protein restriction during gestation and lactation caused a slight increase in testosterone levels on PND 21 (111%; *p* > 0.05; Figure 1A(I)). Meanwhile, at PND 44, the animals exhibited a significant decrease in circulating testosterone levels (a 0.43-fold reduction compared with the normoprotein (NP) group; Figure 1A(II)) and the PND120 animals exhibited a nonsignificant decrease in the levels of this steroid hormone (0.81-fold reduction compared with the NP group) (Figure 1 A(III)).

A maternal low-protein diet significantly increased serum aldosterone levels in 21-day-old animals (a 3.55-fold increase compared with the NP group; Figure 1B(I)). Otherwise, PND 44 and 120 animals showed a gradual decrease in the levels of this hormone compared with low-protein (LP) animals at PND 21 (LP PND 44: 0.16-fold decrease compared with LP PND 21; LP PND 120: 0.09-fold decrease compared with LP PND 21). After comparing the LP and NP groups at the same ages, we observed that maternal protein restriction increased serum aldosterone levels at PND 44 (1.38-fold increase compared with the NP group; *p* > 0.05) and decreased aldosterone levels at PND 120 (0.47-fold decrease compared with the NP group; *p* > 0.05), but the difference was not significant (Figure 1B(II,III)).

### 2.2. VEGFr-2 but Not VEGFa Expression is Altered by Maternal Protein Restriction

VEGFa is a growth factor that is secreted as a dimer and plays a variety of functions in angiogenesis and vascular permeability mainly through VEGFr-2. Although VEGFa binds to VEGRr-1 with high affinity, this binding induces only limited downstream signaling. Thus, VEGRr-1 limits the availability of VEGFa to VEGFr-2, restricting the action of this growth factor [34,35]. Furthermore, the VEGFa/VEGFr-2 axis is able to affect the epididymal microenvironment and sperm maturation as the main regulator of angiogenesis in the organ [32]. Therefore, we investigated the effects of maternal protein restriction on VEGFa and VEGFr-2 expression in the epididymis of the offspring.

**Figure 2:**
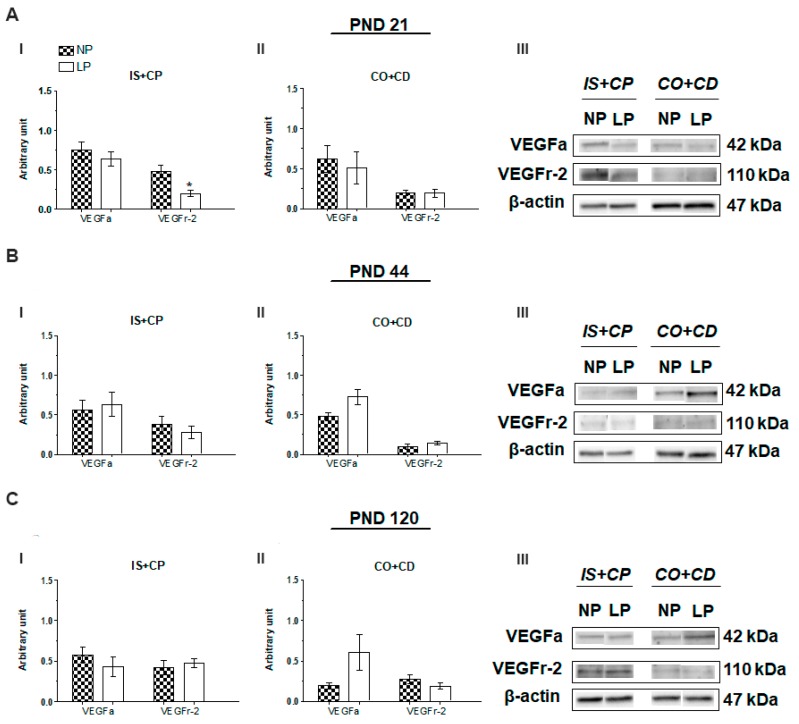
The VEGFa and VEGFr-2 immunoblots. (**A**) Levels of VEGFa and VEGFr-2 in the IS+CP **(I)** and corpus plus cauda (CO+CD) epididymis (**II**) of NP and low-protein (LP) animals on PND 21. Representative blots showing the levels of the VEGF, VEGFr-2 and β-actin proteins (70 µg of protein) in 21-day animals (**III**). (**B**) Levels of VEGFa and VEGFr-2 in the IS+CP (**I**) and CO+CD epididymis (**II**) of NP and LP animals on PND 44. Representative blots showing the levels of the VEGF, VEGFr-2, and β-actin proteins (70 µg of protein) in 44-day animals (**III**). (**C**) Levels of VEGFa and VEGFr-2 in the IS+CP (**I**) and CO+CD epididymis (**II**) of NP and LP animals on PND 120. Representative blots showing the levels of the VEGF, VEGFr-2 and β-actin proteins (70 µg of protein) in 120-day animals (**III**). Data are presented as means ± S.E.M. * *p* < 0.05, Mann-Whitney test.

Although maternal protein restriction during gestation and lactation increased serum aldosterone levels in 21-day-old animals (Figure 1B(I)), the epididymal expression of VEGFa was unchanged at all of the analyzed ages (Figure 2A–C). Notably, we observed a significant decrease in VEGFr-2 expression in the initial segment plus caput (IS+CP) epididymis of PND 21 animals (0.42-fold decrease compared with the NP group) (Figure 2A(I)).

### 2.3. A Maternal Low-Protein Diet Decreases the Microvascular Density (MVD) on PND 21 and 44

**Figure 3:**
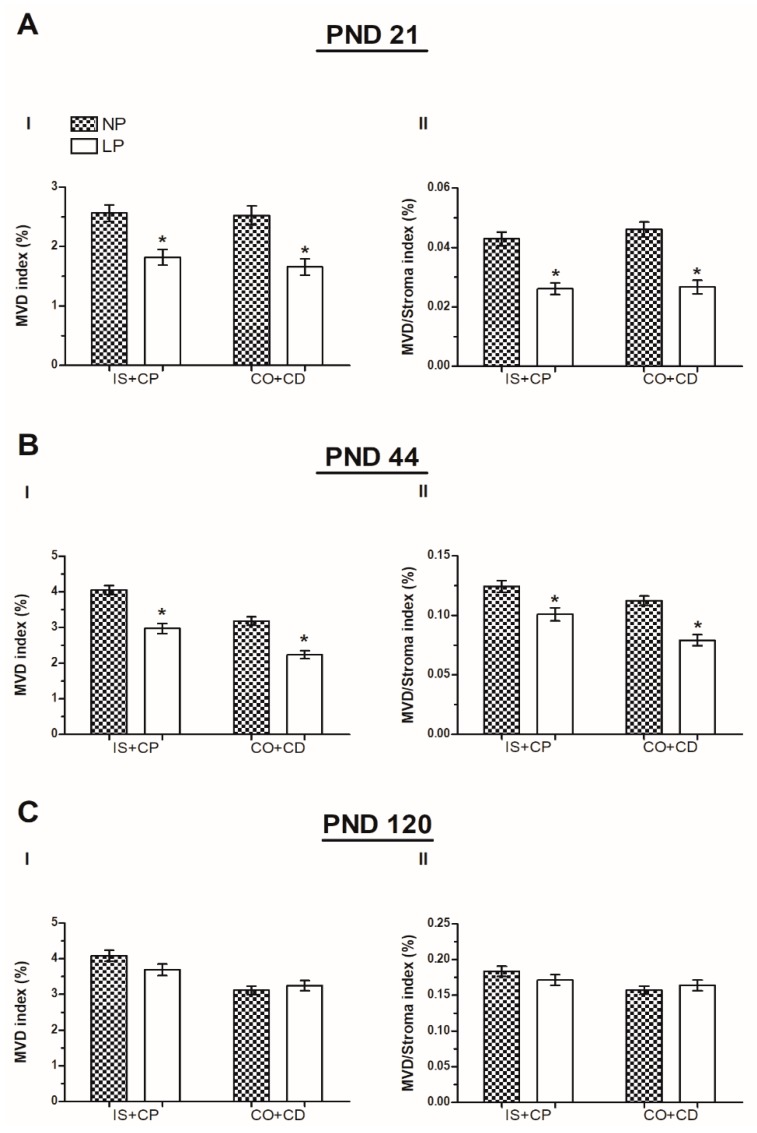
The microvascular densities. (**A**) microvasculature density (MVD) index (**I**) and MVD/Stroma index (**II**) in the IS+CP and CO+CD of 21-day-old animals. (**B**) MVD index (**I**) and MVD/Stroma index (**II**) in the IS+CP and CO+CD of 44-day-old animals. (**C**) MVD index (**I**) and MVD/Stroma index (**II**) in the IS+CP and CO+CD of 120-day-old animals. Data are presented as means ± S.E.M. * *p* < 0.05, Mann-Whitney test.

The MVD index and the MVD/Stroma index were used to analyze the epididymis microvasculature; these values revealed a significant reduction in the blood supply of the IS+CP (the MVD index was decreased 0.71-fold decreased compared with the NP group; the MVD/Stroma index was decreased 0.72-fold compared with the NP group) and corpus plus cauda (CO+CD) (MVD index: 0.66-fold decrease compared with the NP group; MVD/Stroma index: 1.7-fold decrease compared with the NP group) in LP animals compared to the NP group on PND 21 (Figure 3A(I,II)). This reduction was maintained until PND 44 (IS+CP MVD index: 0.72-fold decrease compared with the NP group; IS+CP MVD/Stroma index: 0.83-fold decrease compared with the NP group; CO+CD MVD index: 0.7-fold decrease compared with the NP group; CO+CD MVD/Stroma index: 0.73-fold decrease compared with the NP group; Figure 3B(I,II)). However, this difference was no longer observed at PND 120, and the MVD index and the MVD/Stroma index of LP animals were similar to animals whose mothers consumed the normoprotein diet (Figure 3C(I,II)).

### 2.4. The Impact of the Maternal Low-Protein Diet on AQP1 and AQP9 Immunolocalization

**Figure 4:**
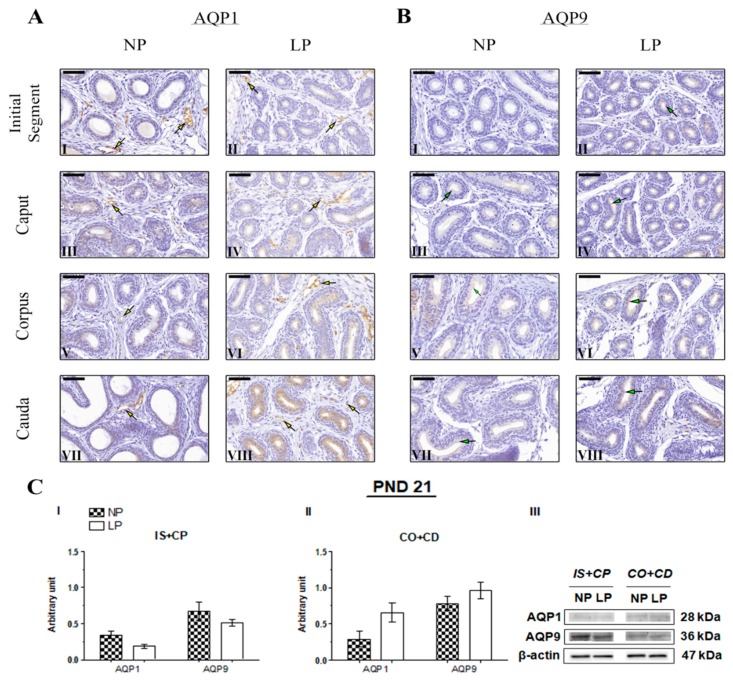
Expression and immunolocalization of aquaporins (AQP)1 and AQP9 in the epididymis of 21-day-old animals. (**A**) Immunoreactivity for AQP1 in endothelial cells of vascular channels in the initial segment (**I** and **II**), caput (**III** and **IV**), corpus (**V** and **VI**), and cauda (**VII** and **VIII**) of NP and LP animals (yellow arrows). (**B**) Immunoreactivity for AQP9 in the stereocilia of epididymis principal cells in the initial segment (**I** and **II**), caput (**III** and **IV**), corpus (**V** and **VI**), and cauda (**VII** and **VIII**) of NP and LP animals (green arrows). Bar = 50 µm. (**C**) Extracts obtained from individual animals were used for a densitometry analysis of the levels of proteins in the initial segment plus caput (**I**) and corpus plus cauda (**II**) following normalization to the housekeeping protein β-actin. Representative blots showing the levels of the AQP1, AQP9 and β-actin proteins (**III**, right panel). Data are presented as means ± S.E.M. and were analyzed using the Mann-Whitney test.

**Figure 5:**
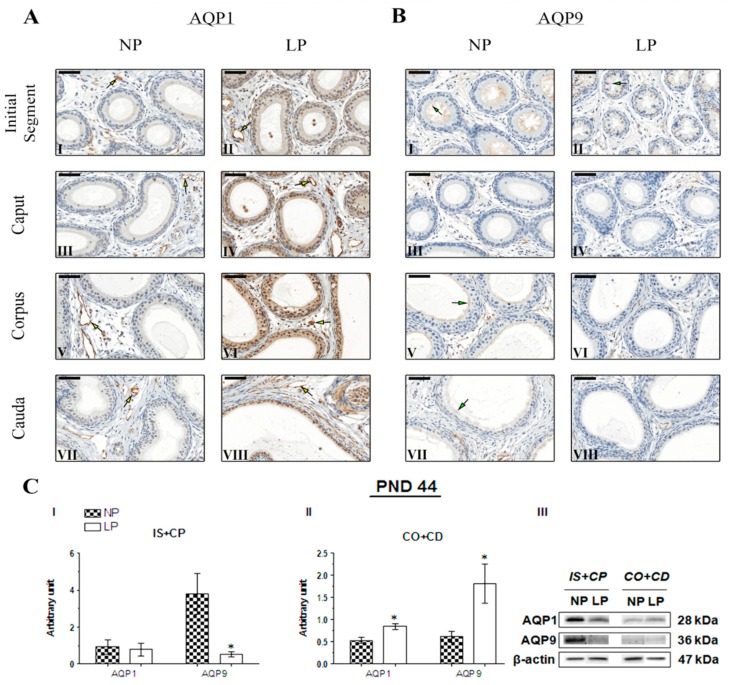
Expression and immunolocalization of AQP1 and AQP9 in the epididymis of 44-day-old animals. (**A**) Immunoreactivity for AQP1 in endothelial cells of vascular channels in the initial segment (**I** and **II**), caput (**III** and **IV**), corpus (**V** and **VI**) and cauda (**VII** and **VIII**) of NP and LP animals (yellow arrows). (**B**) Immunoreactivity for AQP9 in the stereocilia of epididymis principal cells in the initial segment (**I** and **II**), caput (**III** and **IV**), corpus (**V** and **VI**) and cauda (**VII** and **VIII**) of NP and LP animals (green arrows). Bar = 50 µm. (**C**) Extracts obtained from individual animals were used for a densitometry analysis of the levels of the proteins in the initial segment plus caput (**I**) and corpus plus cauda (**II**) following normalization to the housekeeping protein β-actin. Representative blots showing the levels of the AQP1, AQP9 and β-actin proteins (**III**, right panel). Data are presented as means ± S.E.M. * *p* < 0.05, Mann-Whitney test.

**Figure 6:**
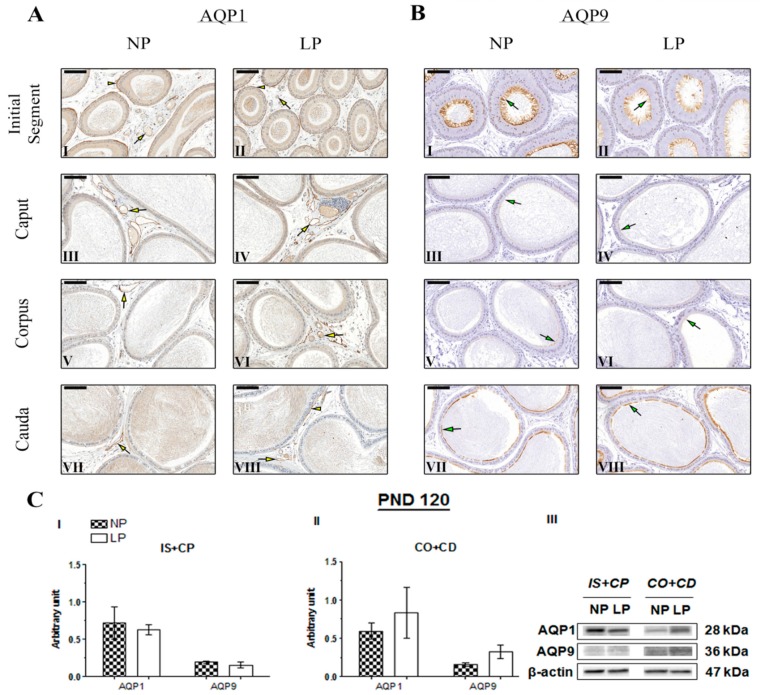
Expression and immunolocalization of AQP1 and AQP9 in the epididymis of 120-day-old animals. (**A**) Immunoreactivity for AQP1 in endothelial cells of vascular channels in the initial segment (**I** and **II**), caput (**III** and **IV**), corpus (**V** and **VI**), and cauda (**VII** and **VIII**) of NP and LP animals (yellow arrows). AQP1 immunostaining was also observed in the peritubular muscle cells of the initial segment in NP and LP animals (**I** and **II**) and in the peritubular muscle cells of the cauda in LP animals (**VIII**) (yellow arrowheads). (**B**) Immunoreactivity for AQP9 in the stereocilia of epididymis principal cells in the initial segment (**I** and **II**), caput (**III** and **IV**), corpus (**V** and **VI**), and cauda (**VII** and **VIII**) of NP and LP animals (green arrows). Bar = 50 µm. (**C**) Extracts obtained from individual animals were used for a densitometry analysis of the levels of the proteins in the initial segment plus caput (**I**) and corpus plus cauda (**II**) following normalization to the housekeeping protein β-actin. Representative blots showing levels of the AQP1, AQP9, and β-actin proteins (**III**, right panel). Data are presented as means ± S.E.M. and were analyzed using the Mann-Whitney test.

No studies have performed AQP1 immunostaining in the epididymis of young animals. Therefore, this study is the first to assess AQP1 immunolocalization in the epididymis of 21- and 44-day-old rats, as well as AQP9 immunolocalization at PND 44. The immunolocalization pattern of AQP1 was the same in PND 21 and 44 animals, and no differences were observed between the NP and LP groups. In these animals, AQP1 staining appeared on endothelial cells of vascular channels throughout the epididymis (Figure 4A and Figure 5A, respectively). At PND 120, AQP1 was observed in endothelial cells of vascular channels throughout the epididymis in both the NP and LP groups (Figure 6A), as well as in the peritubular muscle cells of the initial segment (Figure 6A(I,II)); these findings are consistent with previous studies investigating the role and immunolocalization of aquaporins in the epididymis of adult rats [22,25,36,37,38,39]. However, in the PND 120 animals whose mothers were subjected to protein restriction, AQP1 was also observed on smooth muscle cells adjacent to the epididymal duct in the cauda epididymis (Figure 6AV(III)).

Immunolocalization of AQP9 was observed in the stereocilia of epididymis principal cells in NP and LP animals at all investigated ages. AQP9 immunostaining appears weak in the initial segment, weak to moderate in the caput, and intense in the corpus and cauda epididymis of 21-day-old animals [36,40]. No AQP9 immunoreactivity was observed in the initial segment of the epididymis of NP animals on PND 21 (Figure 4B(I)). However, in both groups, the intensity of AQP9 staining was weak in the epididymis caput (Figure 4B(III,IV)), strong in the corpus (Figure 4B(V,VI)), and moderate in the cauda at this age (Figure 4B(VII,VIII), thereby revealing a segment-dependent function. The intensity of AQP9 staining was slightly higher in the epididymal cauda of LP animals compared with NP animals (Figure 4B(VIII)).

In the 44-day-old NP animals, strong labeling for AQP9 was observed in stereocilia of principal cells in the initial segment (Figure 5B(I)). In the caput epididymis, the staining disappeared (Figure 5B(III)), and was only intensified in the corpus and cauda of the organ (Figure 5B(V,VII)). In PND 44 LP animals, slightly less intense AQP9 staining was observed in the initial segment than in the NP group (Figure 5B(II)), and staining was not observed in caput, corpus, and cauda epididymis (Figure 5B(IV, VI and VIII)). However, AQP9 expression in these regions was also observed and quantified using Western blotting, probably due to the higher sensitivity of the technique.

At PND 120, NP animals showed intense AQP9 staining in the initial segment and cauda epididymis (Figure 6B(I,VII)), corroborating the data from literature [25,37,38,39,40]. At PND 120, slightly less intense AQP9 staining was observed in the initial segment of LP animals than in NP animals (Figure 6B(II)), whereas no differences were observed in the intensity of AQP9 staining in the caput, corpus, and cauda epididymis between NP and LP animals (Figure 6B(III–VIII)).

### 2.5. The Maternal Low-Protein Diet Changes AQP1 and AQP9 Expression in the Epididymis of the Offspring

Maternal protein restriction during gestation and lactation decreased AQP1 and AQP9 expression in the IS+CP and increased the levels of these proteins in CO+CD epididymis at all ages analyzed. However, these results were only significant for AQP9 in the IS+CP (0.14-fold decrease compared with the NP group) and CO+CP (2.94-fold increase compared with the NP group) and for AQP1 in the CO+CD (1.6-fold increase compared with the NP group) on PND 44. At this age, AQP1 expression was only slightly decreased in the IS+CP (0.84-fold decrease compared with the NP; *p* > 0.05) (Figure 5C(I,II)).

Importantly, although the difference was not significant, AQP1 and AQP9 expression decreased in the IS+CP (0.56-fold decrease in AQP1 levels compared with the NP group; 0.76-fold decrease in AQP9 levels compared with the NP group) and increased in the CO+CD (2.28-fold increase in AQP1 levels compared with the NP group; 1.23-fold increase in AQP9 levels compared with the NP group) at PND 21 (Figure 4C(I,II)). This same pattern of AQP1 and AQP9 expression was observed in 120-day-old animals, both in the IS+CP (0.88-fold decrease in AQP1 levels compared with the NP group; 0.79-fold decrease in AQP9 levels compared with the NP group) and CO+CD (1.42-fold increase in AQP1 levels compared with the NP group; 2.00-fold increase in AQP9 levels compared with the NP group) (Figure 6C(I,II)).

## 3. Discussion

Nutrition during pregnancy is a factor that is capable of activating physiological interactions between the mother and fetus through several mechanisms, including hormonal signaling, causing epigenetic alterations and the regulation of the target tissues of these hormones. These interactions may modify placental efficiency and body growth, metabolism, and fetal organ function, providing the basis for various diseases when the availability of gestational and postnatal nutrients is discordant [6].

Although some effects of protein restriction may be a consequence of direct changes in substrate availability, several others are mediated by hormonal effects. These changes may alter the development of specific fetal tissues during critical periods of development or induce long-lasting changes in hormone secretion or in tissue sensitivity to these hormones [41,42,43].

In the male genital system, the testis and epididymis are the main targets of androgen action, as testosterone is essential for the maintenance of the epididymal structure and functions [44,45]. Furthermore, higher androgen concentrations are detected in the epididymal lumen than in the circulation [46]. In the present study, a low-protein diet decreased the serum testosterone level on PND 44 without significantly altering the level of this hormone on PND 21. Importantly, the maintenance of testosterone levels on PND 25, followed by a significant decrease in the concentration of this hormone on PND 70 in animals whose mothers were subjected to protein restriction during pregnancy and lactation, has been reported [12], confirming the role for protein deficiency at the beginning of development in the decreased activity of the pituitary-gonadal axis throughout the animal’s life.

For many years, aldosterone, which is synthesized by the adrenal glands, was considered a “renal hormone”, since has important functions in the cortex of the organ [47,48]. However, aldosterone also exerts extrarenal effects. In the male genital system, more specifically in the epididymis, several studies have documented the influence of aldosterone on the absorption of fluids from the epididymal lumen, mostly in the epididymis caput but also throughout the organ. Thus, aldosterone contributes to one of the main epididymal functions—the concentration of spermatozoa [27,28,29].

Maternal protein restriction is the most widely used model to investigate the effects of fetal programming on the development of chronic diseases in adulthood, particularly hypertension [49]. Based on accumulating evidence, aldosterone substantially contributes to the establishment of high blood pressure in the offspring whose mothers consumed a low-protein diet during gestation [49,50,51]. In this context, studies have observed and correlated hypertension and low birth weight with increased circulating aldosterone levels in maternal protein restriction models [52,53]; these findings are consistent with the increased aldosterone levels observed in LP animals on PND 21 (data on pups’ weights at birth are shown in Table S1: Body weights of male and female offspring at birth).

Considering the adrenal glands, serum VEGF can increase aldosterone availability in the circulation through its action on epithelial cells in the glomerular zone or throughout the endothelial layer, both of which occur in a renin-independent manner [54]. Furthermore, aldosterone is able to modulate VEGF expression or serum concentrations, depending on the cell type or tissue analyzed [55,56,57]. To date, no studies have examined the influence of this mineralocorticoid on the expression of VEGF in the epididymis and its consequences on the vasculature of this organ.

The decrease in VEGFr-2 expression induced by increased aldosterone concentrations has already been observed in rat progenitor endothelial cells [55] and in human umbilical vein endothelial cell cultures [58]. Thus, the increased levels of this mineralocorticoid observed in 21-day-old LP animals might be related to the decrease in VEGFr-2 expression in the IS+CP epididymis of these animals. Additionally, the caput epididymal region is more responsive to the actions of aldosterone [27,28,29], consistent with the lower expression of VEGFr-2 observed in the IS+CP, but not in the CO+CD epididymis.

The lower VEGFr-2 expression observed in the IS+CP epididymis and the decreased epididymal MVD index observed in 21-day LP animals revealed that a maternal low-protein diet altered the epididymal structure and function in the offspring without altering VEGF expression, even if the difference in the vascular pattern was not maintained until adulthood. The epididymis microvascular pattern was influenced in a period of epididymal postnatal differentiation in which the peak of proliferative activity of the initial segment cells occurs, in addition to the complete formation of the blood-epididymal barrier [59]. At this age, adequate blood supply is imperative to promote the regulated transport of metabolites, hormones, and nutrients that reach the epididymis during its postnatal development to maintain the specificity of the microenvironment [30,31,60,61].

More recently, lower levels of testosterone have been associated with increased levels of Nestin, a class VI intermediate filament protein related to neovascularization, and this negative correlation increases the epididymal vasculature in rats [62]. In the present study, despite the reduced MVD index in LP animals at PND 44, we observed a significant decrease in serum testosterone levels that might be a first sign of the substantial increase in the microvasculature of protein-restricted animals, thus nearing the MVD values of LP and NP animals on PND 120.

AQP9 is the predominant aquaporin in the epididymis, playing an important role in the dynamics of reabsorption/secretion and solute transport throughout the organ [25,36,63,64]. Castrated rats show a decrease in epididymal AQP9 expression that is reversed by the administration of testosterone [65], confirming the roles of androgens, such as testosterone and its main metabolite, dihydrotestosterone, in modulating AQP9 expression in the epididymis, particularly in the initial segment [37]. Testicular fluid reaching the epididymal lumen is enriched in testosterone and, despite the high level of this hormone in the whole organ, substantially higher concentrations of dihydrotestosterone are observed in the caput epididymis, indicating the greater sensitivity of this region to variations in androgen concentrations [66]. Because AQP9 expression is modulated by testosterone and the caput epididymal region is more sensitive to changes in the levels of this hormone [65,67], the lower expression of AQP9 observed in the IS+CP epididymis of LP animals at PND 44 might be related to the decreased serum testosterone levels observed in LP animals at this age.

A marked increase in sperm and protein concentrations occurs during spermatozoa transit through the epididymis, indicating an elevation in fluid reabsorption, particularly in the initial segment and caput epididymis. The concentration of proteins in the luminal fluid increases from 4 mg/mL in the initial segment to a maximum of 50–60 mg/mL in the distal cauda [20,25,68,69]. The decrease in AQP9 expression observed in the IS+CP of LP animals on PND 44 may have resulted in lower water uptake in this region, allowing more water to reach the corpus and cauda epididymis. In the epididymal cauda, water reabsorption still occurs to provide more efficient space for the action of imobiline, a protein secreted by epididymis principal cells, whose main function is to immobilize the spermatozoids while they are stored in this region [36,70]. We postulate that the increased AQP9 expression observed in the CO+CD of PND 44 LP animals represents a compensatory mechanism to remove the excess water from the lumen in an attempt to preserve epididymal functions and the balanced intraluminal environment.

The increased AQP9 expression observed in the CO+CD may drain the excess water that was not absorbed by IS+CP and result in increased water in the intertubular space, leading to the appearance of edema in the epididymal cauda. In the epididymis, water is transported from the lumen via AQP9, which is expressed in the stereocilia of principal cells, and then removed from the intertubular space by the action of AQP1, which is expressed on the endothelial cells of the vascular channels throughout the organ [36]. Therefore, the increase in AQP1 expression observed in the CO+CD of PND 44 LP animals might be a physiological strategy to eliminate the excess water from the whole organ.

Although the decrease in serum testosterone levels was not significant in LP animals at PND 120, it seems to have been quite sufficient enough to directly or indirectly alter the expression of aquaporins in the epididymides of these animals in the same manner to the LP animals on PND 44. In PND 21 LP animals, testosterone levels were slightly increased by maternal protein restriction, indicating that animals at this age are responsive to the alterations in the expression of AQP1 and AQP9 in a hormone-independent manner, since the expression of AQP9 does not appear to exclusively depend on the higher levels of androgens that are achieved during and after sexual maturation in rats [65].

Some studies have shown that both aldosterone injections and aldosterone replacement in adrenalectomized rats are able to decrease AQP1 expression in the cochlea of guinea pig [71,72] and in the renal inner medulla of rats [73], respectively. As the caput epididymal region is more responsive to the actions of aldosterone [27,28,29], the increase of the aldosterone level in LP animals at PND 21 and 44 may be involved in the lower AQP1 expression observed in the IS+CP of these animals. In PND 120 LP animals, aldosterone levels were decreased by maternal protein restriction, thereby indicating that the alterations in the expression of AQP1 occurred in an aldosterone-independent manner in animals at this age. 

Notably, although the decrease in AQP9 expression observed in the IS+CP together with the increase in AQP1 and AQP9 expression observed in the CO+CD were only significant for LP animals at PND 44, these alterations were also observed in PND 21 and PND 120 animals whose mothers were subjected to protein restriction during gestation and lactation. Our data support the hypothesis that the low-protein diet altered the expression of these proteins in the epididymis of the offspring, which led to the alterations in epididymal functions related to sperm motility, viability, and concentration, as previously observed by other authors using this experimental model [13,15].

## 4. Materials and Methods 

### 4.1. Animals

Twenty male and thirty-eight female Wistar rats (45 days old) were purchased from the Central Biotherium, Institute of Biosciences/Campus of Botucatu, UNESP—São Paulo State University (Botucatu, Sao Paulo, Brazil). All animals were maintained in polyethylene cages (43 × 30 × 15 cm) with an autoclaved pine shaving substrate under controlled light (12-h light/dark cycle) and temperature (22 ± 1 °C) conditions. The animals were provided with filtered water and a solid diet for rodents ad libitum. The experimental procedures were performed in accordance with the Ethical Principles on Animal Experimentation adopted by the Brazilian College of Animal Experimentation (COBEA) and were approved by the Ethics Committee on Animal Experimentation (EAEC) of the Institute of Biosciences of Botucatu with number 797-CEUA (01/22/2016).

### 4.2. Experimental Design

**Figure 7:**
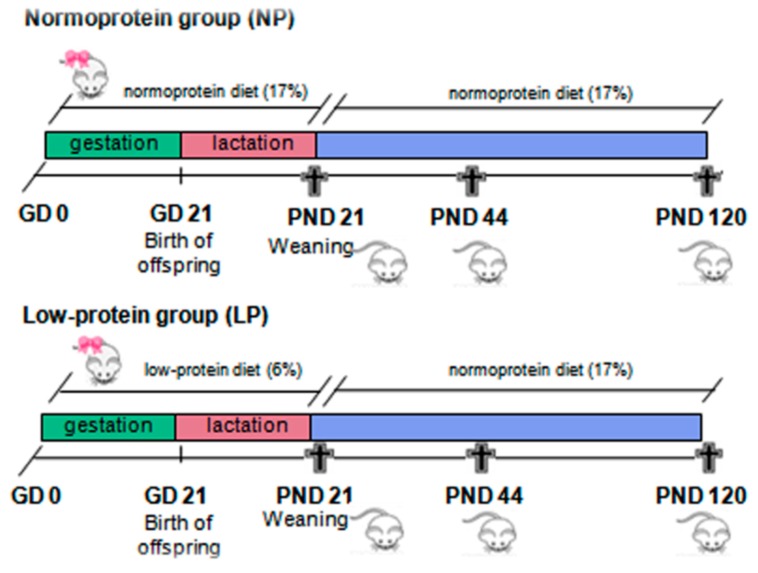
The experimental design. Pregnant females were fed with low-protein diet (LP group) or normoprotein diet (NP group) ad libitum during gestation and lactation from GD 0 until PND 21. After weaning, male pups from both groups received the standard diet for rodents until the ages of 21, 44, and 120 days.

At an age of 95 days, two receptive females were placed in maternity boxes with one male rat overnight for mating. Early the next morning, vaginal smears were collected to evaluate the presence of spermatozoa, confirming the pregnancy; this time point was designated gestational day (GD) 0. Pregnant females were randomly divided into two groups (*n* = 19): A low-protein (LP) group, in which mothers were fed a low-protein diet (6%) ad libitum during gestation and lactation, and a normoprotein (NP) group, in which mothers were fed a normoprotein diet (17%) ad libitum during gestation and lactation. Normoprotein and low-protein diets were offered to the indicated groups from GD 0 until offspring were weaned at PND 21. Only eight pups per litter, preferably males, were maintained with each mother to ensure equal availability of nourishment. After weaning, the LP and NP male pups received the standard diet for rodents until the ages of 21 (NP, *n* = 17; LP, *n* = 19), 44 (NP, *n* = 12; LP, *n* = 10) and 120 (NP, *n* = 13; LP, *n* = 12) days, when they were euthanized and the blood and epididymides were collected (Figure 7). The ages of 21, 44, and 120 days were based on three different phases of postnatal epididymal development: The peak of cell differentiation, the final period of epididymis differentiation, and the beginning of its expansion in a well-differentiated adult epididymis, respectively.

### 4.3. Hormonal Assay

Animals were anesthetized by induced narcosis in a CO2 chamber, euthanized by decapitation, and the blood was collected by cervical vessel rupture. Blood serum was obtained by centrifugation at 14,000 rpm for 20 min at 4 °C. Serum samples were assayed for testosterone and aldosterone levels using chemiluminescence with specific kits provided by “Beckman Coulter, Inc.” (Brea, CA, USA) and “DiaSorin Inc.” (Stillwater, MN, USA), respectively. The lower limit of detection for testosterone was 10 ng/dL and the lower and the higher limits of detection for aldosterone was 0.97 ng/dL and 100 ng/dL, respectively. All samples were assayed at the same time to prevent interassay variation.

### 4.4. Immunohistochemistry

After euthanasia, the right epididymides were collected, dissected, fixed with 10% buffered formalin (0.1 M phosphate buffer, pH 7.3) for 24 h, then washed, dehydrated in a graded series of ethanol solutions, diaphanized in N-butyl alcohol, and embedded in paraplastic (Paraplast Plus, St. Louis, MO, USA). The epididymides were cut into 5-μm-thick sections using a LEICA RM 2165 micrometer (Leica Biosystems, Nußloch, Germany). Four blocks were cut from each group (LP and NP) at each age; the blocks from 21-day-old animals were cut into serial sections, while the blocks from 44- and 120-day-old animals were cut into semi-serial sections.

For immunohistochemistry, epididymal sections from LP and NP animals (*n* = 4 animals/group at each age) were deparaffinized in the oven (40 min at 60 °C), followed by a 15 min incubation in xylene and hydration with decreasing concentrations of ethanol. Antigen retrieval was performed in 0.01 M sodium citrate buffer, pH 6.0, in a microwave for 20 min (4 × 5 min). Afterward, endogenous peroxidases were blocked (H_2_O_2_, 0.3% in methanol) and then the tissues were incubated with 3% BSA for 1 h. The epididymal sections were incubated overnight at 4 °C with a 1:200 dilution of the following primary antibodies in 1% BSA: Anti-AQP1 (Millipore, Temecula, CA, USA) and anti-AQP9 (Alpha Diagnostic, San Antonio, TX, USA). On the next morning, sections were washed with PBS and then incubated with 1:200 dilutions of a peroxidase-conjugated anti-Rb secondary antibody (Sigma, St. Louis, MO, USA) for AQP1 and biotinylated anti-Rb secondary antibody (Sigma, St. Louis, MO, USA) for AQP9 both in 1% BSA for 2 h. The sections labeled with biotinylated antibodies were incubated with the ABC complex (ABC Vectastain^®^ kit, Burlingame, CA, USA) for 45 min and subsequently washed with PBS buffer. The immunoreactive components were reacted with diaminobenzidine (DAB; Sigma, St. Louis, MO, USA) and the slides were counterstained with hematoxylin. Finally, the slides were scanned using a 3D Histech Pannoramic MIDI and analyzed and photographed using the Pannoramic Viewer program.

### 4.5. Western Blot

The left epididymis of five animals from each group (LP and NP) at the ages of 21, 44 and 120 days were divided into initial segment plus caput (SI+CP) and corpus plus cauda (CO+CD). Samples were homogenized at 4 °C with RIPA lysis buffer (Bio-Rad, Hercules, CA, USA) supplemented with a protease inhibitor cocktail (Sigma, St. Louis, MO, USA). The homogenate was then centrifuged at 14,000 rpm for 20 min at 4 °C to remove the cell debris, and the supernatant was collected. Total protein concentrations were quantified using the Bradford colorimetric method [74]. Then, aliquots of 70 µg of protein were treated with 1.5× Laemmli buffer and the proteins were then separated by 4–15% polyacrylamide gel electrophoresis (SDS-PAGE) for 90 min at 120 V. Following electrophoresis, proteins were electrotransferred to a nitrocellulose membrane in a wet system at 350 mA. Nonspecific protein binding was blocked with 3% milk (Molico^®^) in TBS-T buffer for 1 h at room temperature. Membranes were incubated overnight at 4 °C with the following primary antibodies diluted in TBS-T: Anti-AQP1 (1:800 dilution; Millipore, Temecula, CA, USA), AQP9 (1:500 dilution; Alpha Diagnostic, San Antonio, TX, USA), VEGFa (1:1000 dilution; Santa Cruz, Santa Cruz, CA, USA), VEGFr-2 (1:1000 dilution; Santa Cruz, Santa Cruz, CA,USA) and β-actin (1:800 dilution; Santa Cruz, Santa Cruz, CA, USA). On the next morning, membranes were washed with TBS-T buffer and incubated with an anti-Rb secondary antibody for VEGFa (1:2000 dilution; Sigma, St. Louis, MO, USA), AQP1 and AQP9 (1:5000 dilution; Sigma, St. Louis, MO, USA), anti-Ms secondary antibody for VEGFr-2 (1:5000 dilution; Abcam, Cambridge, UK) and anti-goat secondary antibody (1:6000 dilution; Sigma, St. Louis, MO, USA) diluted in TBS-T for 2 h, and then washed with TBS-T buffer. Thereafter, the immunoreactive bands were revealed with a luminescence kit (Amersham ECL™ Western Blotting Detection Reagent Select) from GE Healthcare^®^ and subjected to semiquantitative analysis with optical densitometry using ImageJ analysis software for Windows. The values obtained for each band of AQP1, AQP9, VEGFα, and VEGFr-2 were normalized to the β-actin density and the data are presented as means ± S.E.M. The optical densitometry index (% band intensity) was used to represent immunoblotting data.

### 4.6. Microvascular Density (MVD) Determination

The MVD was determined by adapting the procedures described by Reference [75]. Briefly, epididymal sections from LP and NP animals (*n* = 4 animals/group at all ages) that had been immunostained with the monoclonal anti-AQP1 antibody were evaluated using a stereological analysis [76] to estimate the number of microvessels in the initial segment plus caput and corpus plus cauda epididymis. Data are presented as the percentage of microvessels per total area analyzed.

### 4.7. Statistical Analysis

Comparisons between LP and NP groups at all analyzed ages were performed using the Student’s t-test for parametric data and the Mann-Whitney test for nonparametric data. Data are presented as means ± S.E.M., and differences were considered statistically significant when *p* < 0.05. GraphPad Prism^®^ software (version 5.00, Graph Pad, Inc., San Diego, CA, USA) was used to perform the statistical analyses. 

## 5. Conclusions

The low-protein diet increased aldosterone levels and decreased VEGFr-2 expression in the initial segment and caput epididymis during an important period of postnatal epididymal development, thereby influencing the epididymal vascularization pattern and fluid transport and potentially interfering with the structure and function of the organ. Furthermore, the pups whose mothers had limited protein intake showed a decrease in AQP9 expression in the initial segment and caput and an increase in AQP1 and AQP9 expression in the corpus and cauda, which potentially led to alterations in epididymal functions related to sperm motility, viability, and concentration. Based on our findings, maternal protein restriction during gestation and lactation permanently altered hormone levels and the expression of proteins important for the correct development and proper function of the epididymis in the developing organism.

## Supplementary Materials

Supplementary materials can be found at http://www.mdpi.com/1422-0067/20/3/469/s1. Table S1: Body weight of male and female offspring at birth.

## Abbreviations

NP	Normoprotein
LP	Low-protein
PND	Postnatal day
AQP	Aquaporins
VEGFa	Vascular endothelial growth factor
VEGFr-2	Vascular endothelial growth factor receptor 2
MVD	Microvasculature density
IS+CP	Initial segment plus caput
CO+CD	Corpus plus cauda
