# Supplementary material for: Maternal Protein Restriction Differentially Alters the Expression of AQP1, AQP9 and VEGFr-2 in the Epididymis of Rat Offspring
Source: Int J Mol Sci. 2019 Jan 22;20(3):469. doi: 10.3390/ijms20030469 (PMC6387270; doi:10.3390/ijms20030469)
Supplement: Supplementary file 1 [file ijms-20-00469-s001.pdf]

Table 2. Body weight of male and female offspring at birth.

Parameters	Male		Female	
	**NP**	**LP**	**NP**	**LP**
Number of pups	5.29 ± 0.43	5.47 ± 0.52	5.71 ± 0.62	5.58 ± 0.51
Body weight (g)	6.33 ± 0.12	5.79 ± 0.10 *	5.87 ± 0.09	5.79 ± 0.10 *

NP, N = 17 litters/group. LP, N = 19 litters/group. Data are expressed as the mean ± S.E.M., * $p < 0.05$ vs. NP. Test t and Mann-Whitney for parametric and non-parametric data, respectively.
